# Using the Adaptive Leadership Framework for Chronic Illness to Design a Palliative and Collaborative Intervention

**DOI:** 10.1093/geroni/igab046.648

**Published:** 2021-12-17

**Authors:** Ashley Leak Bryant, Stephanie Becantur, Ya-Ning Chan, Rachel Hirschey, Marianne Baernholdt, Ruth Anderson

**Affiliations:** 1 School of Nursing, Durham, North Carolina, United States; 2 UNC School of Nursing, Chapel Hill, North Carolina, United States; 3 UNC Chapel Hill, Chapel Hill, North Carolina, United States; 4 University of North Carolina at Chapel Hill School of Nursing, Chapel Hill, North Carolina, United States; 5 University of North Carolina at Chapel Hill, UNC Chapel Hill, North Carolina, United States

## Abstract

Our study addresses symptoms and functional management early in acute myeloid leukemia treatment using a PAlliative and Collaborative Care inTervention (PACT) delivered by clinical staff. PACT is an interdisciplinary nurse-led intervention of nursing [RN], occupational therapy [OT], physical therapy [PT] for adults ≥ 60 years of age at time of initial treatment. Finding from our preliminary qualitative work led to use of the Adaptive Leadership Framework for Chronic Illness to guide optimal engagement of the patient in self-management of symptoms and functional activities to address QOL outcomes. We share lessons learned of how the ALFCI was applied to the PACT intervention to empower patients with self-management skills to reduce symptoms and optimize function. In summary, the team and patient identify and address emotional, motivational, attitudinal barriers so that the patient can do the work required for self- management to reduce symptoms and avoid functional decline.

